# Time-Intensity Curve Parameters in Rectal Cancer Measured Using Endorectal Ultrasonography with Sterile Coupling Gels Filling the Rectum: Correlations with Tumor Angiogenesis and Clinicopathological Features

**DOI:** 10.1155/2014/587806

**Published:** 2014-05-13

**Authors:** Yong Wang, Lin Li, Yi-Xiang J. Wang, Ning-Yi Cui, Shuang-Mei Zou, Chun-Wu Zhou, Yu-Xin Jiang

**Affiliations:** ^1^Department of Diagnostic Imaging, Cancer Hospital & Institute, Peking Union Medical College and Chinese Academy of Medical Sciences, Beijing 100021, China; ^2^Department of Imaging and Interventional Radiology, Prince of Wales Hospital, The Chinese University of Hong Kong, Shatin, NT, Hong Kong; ^3^Department of Pathology, Cancer Hospital & Institute, Peking Union Medical College and Chinese Academy of Medical Sciences, Beijing 100021, China; ^4^Department of Diagnostic Ultrasound, Peking Union Medical College Hospital, Peking Union Medical College and Chinese Academy of Medical Sciences, Beijing 100730, China

## Abstract

The primary aim of this study was to investigate the relationship between contrast-enhanced ultrasonography (CEUS) imaging parameters and clinicopathological features of rectal carcinoma and assess their potential as new radiological prognostic predictors. A total of 66 rectal carcinoma patients were analyzed with the time-intensity curve of CEUS. The parameter arrival time (AT), time to peak enhancement (TTP), wash-in time (WIT), enhanced intensity (EI), and ascending slope (AS) were measured. Microvessel density (MVD) was evaluated by immunohistochemical staining of surgical specimens. All findings were analysed prospectively and correlated with tumor staging, histological grading, and MVD. The mean values of AT, TTP, WIT, EI, and AS value of the rectal carcinoma were 10.84 ± 3.28 s, 20.61 ± 5.52 s, 9.78 ± 2.83 s, 28.68 ± 4.67 dB, and 3.20 ± 1.10, respectively. A positive linear correlation was found between the EI and MVD in rectal carcinoma (r = 0.295, P = 0.016), and there was a significant difference for EI among histological grading (r = −0.264, P = 0.007). EI decreased as T stage increased with a trend of association noted (P = 0.096). EI of contrast enhanced endorectal ultrasonography provides noninvasive biomarker of tumor angiogenesis in rectal cancer. CEUS data have the potential to predict patient prognosis.

## 1. Introduction

It is well known that rectal cancer is an important contributor to cancer mortality and morbidity [1]. Angiogenesis, which involves sprouting of endothelial cells to form new vessels and supplying nutriments and oxygen for the tumor cells, is essential for tumor formation, growth, and dissemination [2]. Microvessel density measured by immunofluorescent analysis is used to evaluate tumor angiogenesis activity as standard method, but it is invasive and depending on experience of operators [3, 4]. Noninvasive imaging modalities such as dynamic contrast enhanced magnetic resonance (DCE-MR) [5], perfusion computed tomography [6], and contrast enhanced ultrasound (CEUS) are applied to observe tumor vascularity. Ultrasound is low cost and convenient and no radiation is associated. The second generation of ultrasound contrast agents consists of microbubbles remaining strictly intravascular, leading to CEUS becoming a promising indirect method of evaluating blood flow within functional vessel [7, 8]. Meanwhile, the analysis of time intensity curve (TIC) makes it possible to assess tumor vascularity quantitatively [9].

Recent studies have demonstrated that CEUS perfusion parameters are closely correlated with tumor vascularity in several types of malignancies, such as hepatocellular carcinoma, pancreatic carcinoma, prostate cancer, breast tumors, and gastric carcinoma [10–13]; however, there is limited experience in using CEUS to assess tumor vascularity in rectal cancer. Zhuang et al. [14] demonstrated positive linear correlation between TIC parameters by CEUS and MVD in colorectal tumor, but only two rectal cases were concluded in the study. The value of TIC parameters in assessing tumor vascularity in rectal cancer remained to be investigated.

Some researchers also explored the relationship between DCE-MRI perfusion parameters and prognostic factors in rectal cancer, but results have been conflicting. Oberholzer et al. [15] reported that DCE-MRI parameter correlated with the N category and k21 with the occurrence of distant metastases; Hong et al. [16] reported that Erise was correlated with N stage, and Tp was correlated with histologic grade, while Kim et al. [4] found no correlation between any DCE-MRI perfusion parameters and TN stage. Till now, there have been few reported studies on relationship between CEUS perfusion parameters and prognostic factors in rectal cancer.

Therefore, the purpose of this study was to investigate the correlation of time-intensity curve (TIC) parameters with microvessel density in rectal cancer and we also evaluate the relationship between TIC parameters, MVD, and the standard prognostic variables (tumor stage, lymphatic metastasis, distant metastasis, and histologic grade) to explore the diagnostic value in tumor vascularity and prognostic value of TIC parameters in rectal cancer.

## 2. Materials and Methods

### 2.1. Patients

A total of 66 patients with rectal cancer who underwent endorectal ultrasound (ERUS) and CEUS examinations were involved. All patients had undergone surgery within 1 week after CEUS in our hospital between December 2009 and June 2013. None had undergone radiation or chemotherapy before surgery. Patients with rectal mass who had not been referred for ERUS and CEUS examinations or in whom surgery was not undertaken within one week were not included in this study. ERUS and CEUS examinations were approved by the Hospital Ethics Committee. Each patient was consent informed. All of the patients had solitary lesions. The diagnoses for all 66 lesions were confirmed by surgery and pathology.

### 2.2. ERUS

All ERUS examinations were performed using a Philips iU22 unit (Philips, Bothell, WA, USA). An end-fire type endorectal probe (C5-9 sec) was utilized. Patients stayed in the left lateral decubitus position, prepared with enemas to remove all air, stool, and mucus from the rectum. Instead of the standard water-balloon filling technique, we developed a novel technique in our previous study, where the coupling gel was injected into the rectum directly [17]. The amount of gel used was usually 100–150 mL, depending on filling degree of the rectum, which was to ensure the five layers of the bowel wall and the tumor can be clearly seen. The gel helped the US probe to pass through the tumoral stenosis of rectum, minimized compression and distortion of the lesion, and improved visualization of the rectal wall and tumor. The tumors were evaluated for their size and depth of invasion, echo pattern, and internal vascularity as well as the localization of the rectal wall layers that were disrupted by the tumor.

### 2.3. CEUS

**Figure 1:**
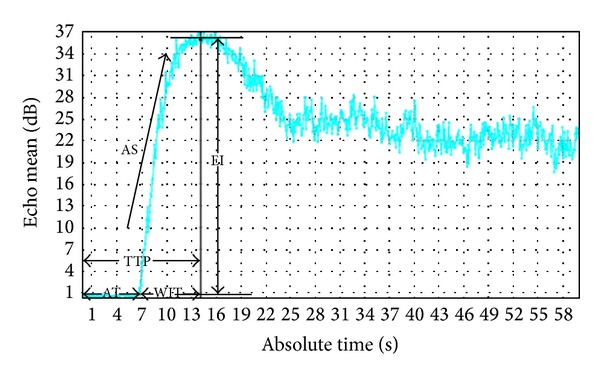
Time-intensity curve (TIC) from region of interest (ROI) within the tumor and the ultrasound TIC parameters. Arrival time (AT), time to peak enhancement (TTP), wash-in time (WIT), enhanced intensity (EI), and ascending slope (AS).

CEUS examination was performed after the ERUS examination. The mechanical index was 0.08–0.11. 2.4 mL contrast agent SonoVue (Bracco, Italy) which was administrated through a forearm vein in bolus through a 20-gauge intravenous cannula within 1 to 2 seconds, followed by a flush of 5 mL of 0.9% normal saline solution. The contrast agent wash in and wash out were recorded for 60 seconds. By using Q lab software (version 5; Philips Medical Systems, Bothell, WA, USA) on the workstation; the region of interest (ROI) of every lesion was manually drawn in the most enhanced region within the tumor on contrast ultrasonographic images and the ROI area was set to 25 mm^2^. The time-intensity curve was reconstructed for each ROI and then arrival time (AT), time to peak enhancement (TTP), wash-in time (WIT), enhanced intensity (EI), and ascending slope (AS) were obtained. The AT was defined as the time from injection until the enhancement. The TTP was defined as the interval from injection to the peak of the time-intensity curve. The WIT was defined as interval from beginning of enhancement to the peak of the enhancement. The EI was defined as peak intensity minus baseline intensity. The AS was defined as the slope rate of ascending curve (Figure 1). All contrast-enhanced ultrasound data were analyzed by two experienced radiologists who were blinded to all clinical and pathological information.

### 2.4. Histopathological Analysis

Histological sections were reviewed by one experienced pathologist without knowledge of the results of the ultrasound findings. The description of the gross specimen and 4 *μ*m thick haematoxylin and eosin-stained histological sections were reviewed. Morphologic prognostic factors including TNM stage and histologic grade were identified according to the World Health Organization classification. Rectal adenocarcinoma are graded by the proportion of fully formed glands seen in microscopic slides and classified as well-differentiated, moderately differentiated, and poorly differentiated. Well differentiated adenocarcinoma shows >95% gland formation. Moderately differentiated adenocarcinoma shows 50–95% gland formation. Poorly differentiated adenocarcinoma is mostly solid with <50% gland formation. To determine MVD, the tissues obtained from the most representative paraffin blocks were mounted on poly-L-lysine-coated slides for immunostaining. The CD34 antibody (Dako, Glostrup, Denmark) was used to label the vascular endothelium cytoplasm. The five most vascularized areas (“hot spot”) with the highest number of microvessel profiles were chosen subjectively from each tumor section by examination under a low power lens (100x magnification); the total number of microvessels labelled with the CD34 antibody was counted for each area under a high power lens (200x magnification). The mean value of the microvessel number was the MVD value of the tumor [18].

### 2.5. Statistical Analysis

All analyses were performed with SPSS version 20 for Windows personal computers (SPSS Inc., Chicago, IL, USA). All data were described as means (SD). Two-tailed *P* values less than 0.05 were considered to indicate a significant difference. Bivariate Pearson correlation analysis was performed to investigate the correlation between CEUS parameters with MVD values and clinicopathologic features.

## 3. Results

A total of 66 patients were included in the study. The age of the patients ranged from 37 to 71 years (mean 55.8 years), with 46 male and 20 female patients. Following the total mesorectal excision (TME) principle, all 66 patients underwent standard rectal cancer resection, including Mile's and Dixon's operations.

### 3.1. Time-Intensity Curve Analysis of Rectal Cancer

**Figure 2:**
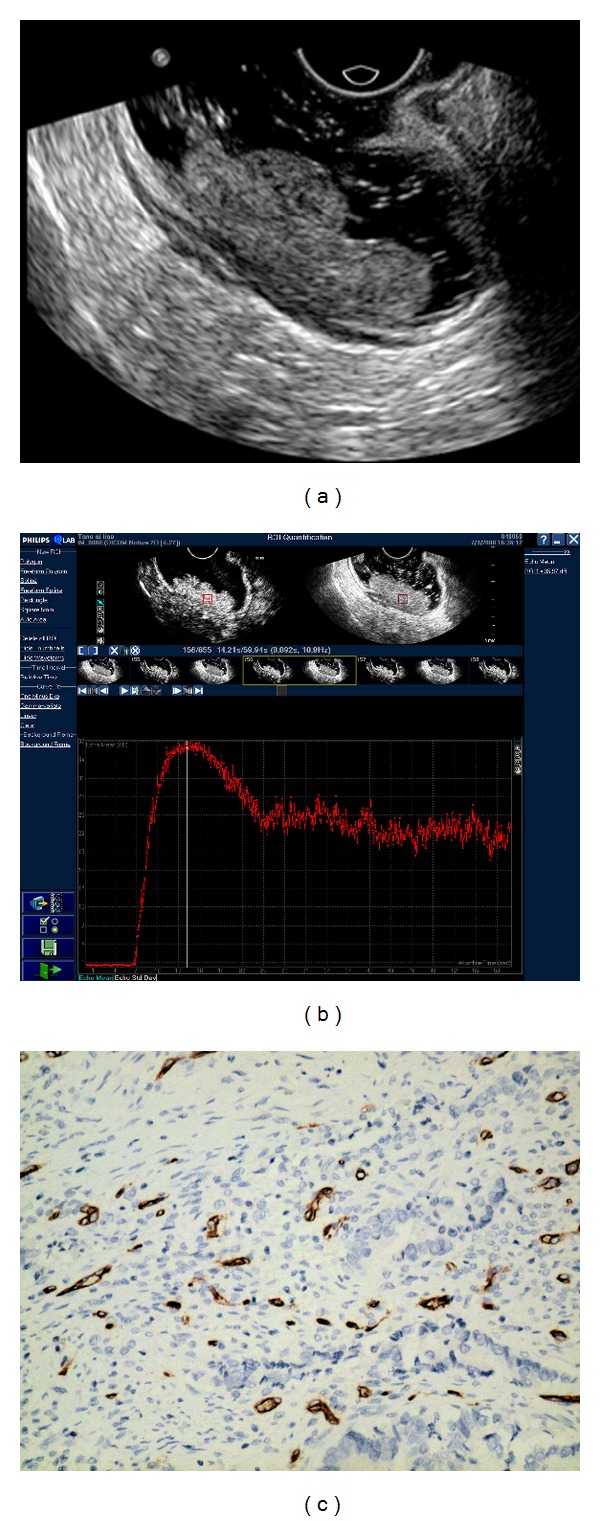
Images of poorly differentiated rectal adenocarcinoma with T2 stage. (a) Endorectal ultrasonography showed that an irregular hypoechoic lesion invaded muscularis propria. (b) Time-intensity curve was obtained from ROI with EI = 36.83 dB, AT = 6.87 s, TTP = 14.57 s, WIT = 7.70 s, and AS = 4.78.  (c) Representative  photomicrographs of Immunohistochemical CD34 staining in the same tumor (200x magnification) showed microvasculature in brown and poorly differentiated rectal adenocarcinoma. The MVD value is 43.

**Figure 3:**
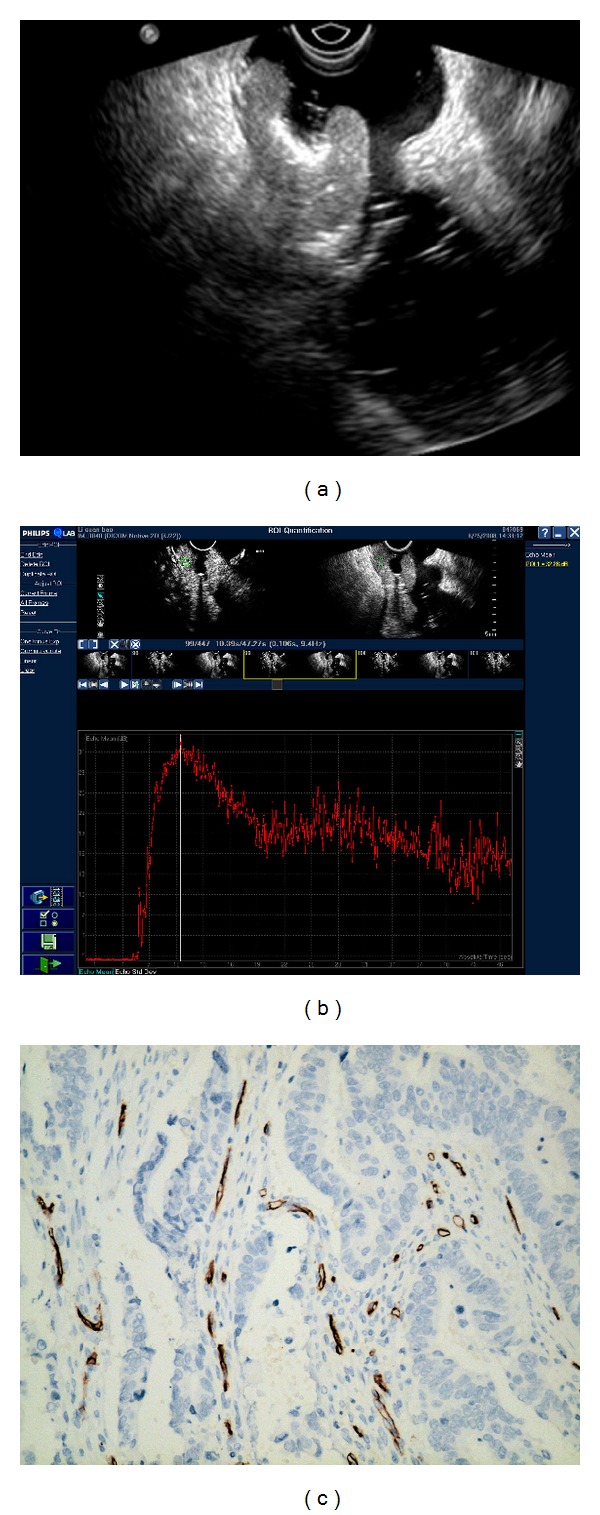
Images of moderately differentiated rectal adenocarcinoma with T3 stage. (a) Endorectal ultrasonography showed an irregular hypoechoic lesion proceeded beyond the muscularis propria and serosa and perirectal fat. (b) Time-intensity curve was obtained from ROI with EI = 29.61 dB, AT = 5.83 s, TTP = 10.49 s, WIT = 4.66 s, and AS = 6.35. (c) Representative photomicrographs of Immunohistochemical CD34 staining in the same tumor (200x magnification) showed microvasculature in brown and moderately differentiated rectal adenocarcinoma. The MVD value is 24.

**Figure 4:**
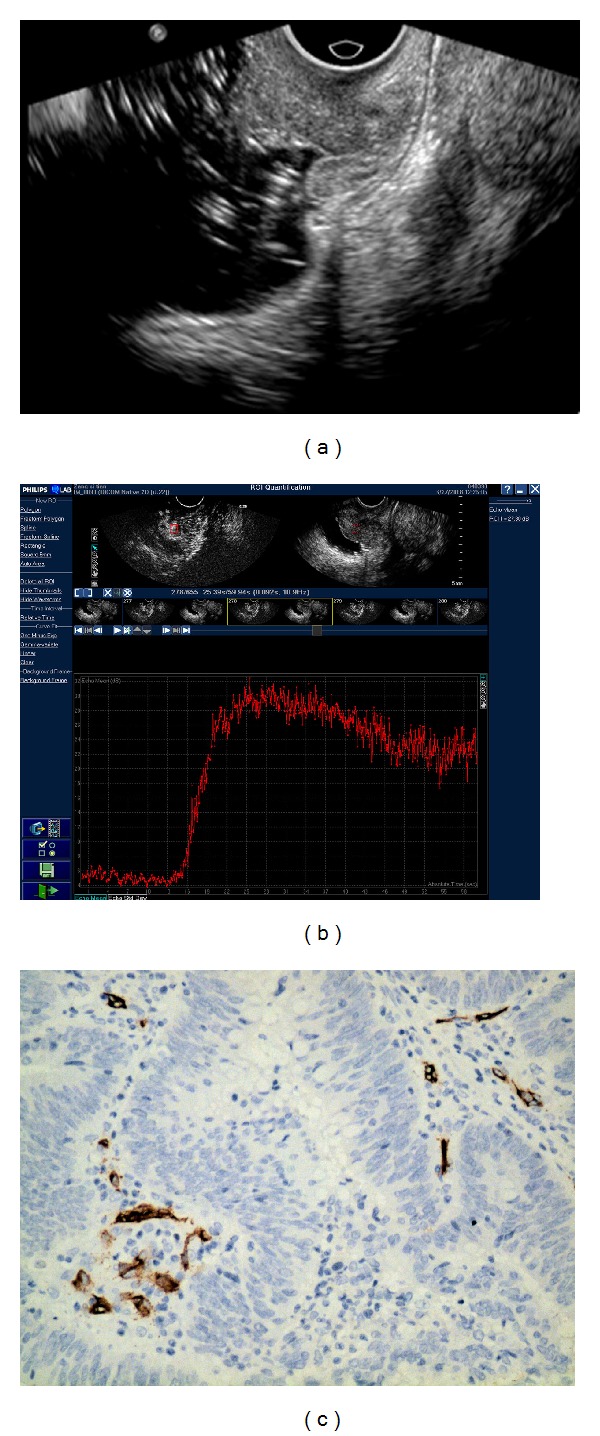
Images of well differentiated rectal adenocarcinoma with T1 stage. (a) Endorectal ultrasonography showed that an irregular hypoechoic lesion invaded both the mucosa and submucosa layer. (b) Time-intensity curve was obtained from ROI with EI = 25.21 dB, AT = 13.01 s, TTP = 25.12 s, WIT = 12.11 s, and AS = 2.08. (c) Representative photomicrographs of Immunohistochemical CD34 staining in the same tumor (200x magnification) showed microvasculature in brown and well differentiated rectal adenocarcinoma. The MVD value is 16.

All of the time intensity curve showed similar enhancement pattern. After the administration of contrast agent, signal intensity increased linearly with time and then reached a plateau then decreased gradually (Figures 2(b), 3(b), and 4(b)). Arriving time ranged 4.35–19.47 sec (10.84 ± 3.28); time to peak enhancement ranged 10.49–34.43 sec (20.61 ± 5.52); wash-in time ranged 4.61–16.69 sec (9.78 ± 2.83); enhanced intensity ranged 18.34–36.83 dB (28.68 ± 4.67). Ascending slope ranged 1.44–6.51 (3.20 ± 1.10).

### 3.2. CEUS Perfusion Parameters and MVD Count

**Figure 5:**
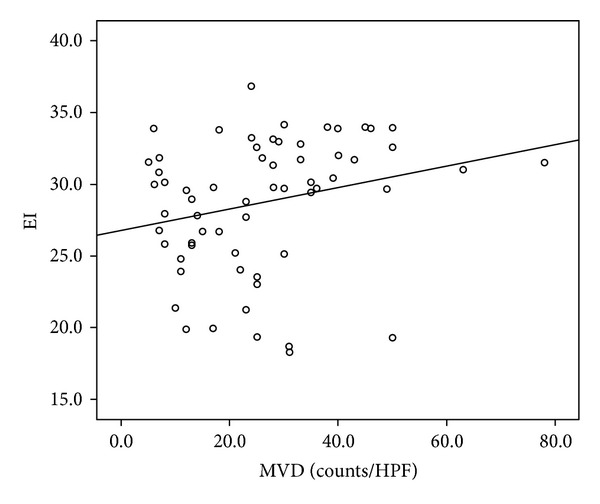
Scatter plots show positive correlations between MVD and EI (*r* = 0.295, *P* = 0.016).

The correlations of CEUS parameters with MVD count are shown in Table 1. The MVD count ranged from 5 to 78 vessels/mm^2^ (26.63 ± 15.23) (Figures 2(c), 3(c), and 4(c)). The enhanced intensity was positively correlated with MVD count (*r* = 0.295, *P* = 0.016) (Figure 5). No statistic differences were found in MVD count with other CEUS parameters (the arriving time, time to peak, ascending slope, and wash-in time) (*P* = 0.179–0.840).

### 3.3. CEUS Parameters and Clinicopathologic Features

**Figure 6:**
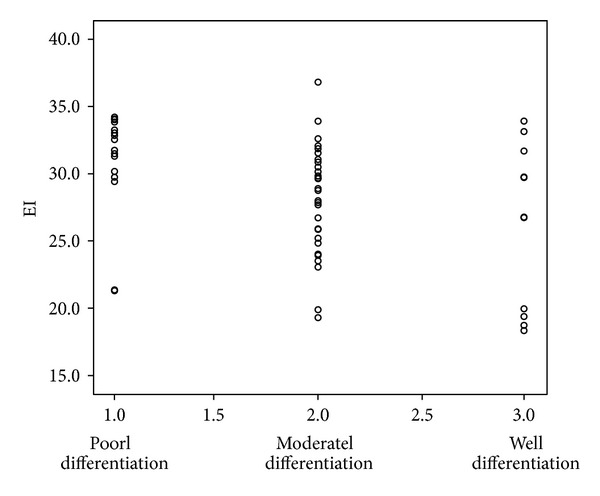
Scatter plots show negative correlations between histologic grade and EI (*r* = −0.264, *P* = 0.007).

All of the 66 lesions were rectal adenocarcinoma. The median diameter for all tumors was 2.5 cm (range 1.8–4.0 cm). Histopathological tumor staging was determined to be T1 in 10, T2 in 12, T3 in 34, (Figures 2(a), 3(a) and 4(a)). and T4 in 10 patients, and N0 in 40 patients, N1 in 10 patients, N2 in 16 patients. 9 patients had hepatic metastases proved in enhanced CT follow-up. The tumors were well-differentiated in 12, moderately-differentiated in 36, and poorly-differentiated in 18 patients. The correlations of CEUS parameters with TNM stage and histologic grade were shown in Table 2. The enhanced intensity was negatively correlated with histologic grade (*r* = −0.264, *P* = 0.007) (Figure 6); poorly differentiated tumors showed higher enhanced intensity compared with well differentiated lesions. It was that noted EI decreased as T stage increased (*P* = 0.096). A trend of association was noted though statistical significance was not reached.

## 4. Discussion

Angiogenesis is a prerequisite factor for tumor growth and metastatic dissemination, and might be indicative for prognosis and treatment option [19–21]. Nowadays, the standard method used for quantitative evaluation of angiogenesis is immunofluorescent analysis of intratumoral microvessel density (MVD), which quantifies the number of vessels per unit volume [22]. However, this method is limited by its following disadvantages. Firstly, tissue samples have to be obtained via invasive biopsy procedures. Secondly, tissue samples only represent a certain area within the tumor. Thirdly, tissue must be obtained repeatedly to monitor changes in tumor angiogenesis. Fourthly, the results are not immediately available for the clinician [23].

Contrast-enhanced ultrasonography (CEUS) is a well accepted and widely available imaging modality in recent years [24–26], because it has overcome the limitations of conventional ultrasonography and created a significant opportunity for visualization of the microcirculation [27]. The second-generation contrast agents (e.g., SonoVue) combined with a low-mechanical index ultrasonographic technique based on nonlinear acoustic effects on interactions with microbubbles make the microbubbles more stable and durable and therefore can facilitate continuous and dynamic observation for a specific period and research of the perfusion of tumor vessels [10]. Furthermore, gray scale CEUS is thought to maximize contrast and spatial resolution, and the diameter of second-generation contrast agent microbubble is only about several micrometers, thereby leading the evolution of CEUS from vascular imaging to imaging of perfused tissue at the microvascular level [27]. Following injection, the bubbles circulate throughout the vascular space and constrictively confined in the microvasculature, which is different from enhanced CT or MR. From time-intensity curve, fractional vascular volume, and flow velocity, relative perfusion rate can be obtained.

Zhuang et al. [14] assessed angiogenesis of colorectal tumor using double contrast enhanced ultrasound (DCEUS). In our experience, contrast-enhanced transabdominal ultrasound is useful in depicting colon cancer, but is not suitable for rectal cancer. In this study, we adapted ERUS for diagnosis of rectal cancer and introduced a novel gels-filling technique. Instead of using a water bath around the probe, the new technique improves the visualization of the rectal cancer and contrast enhanced endorectal ultrasound is less affected by attenuation of the enhancement with depth compared with transabdominal sonography [17].

Recently, increasing positive results regarding the correlation of CEUS parameters with MVD in various cancers have been reported in literatures [10, 13, 28–30]. Although CEUS TIC parameters were investigated in many other malignancies, there are only few studies dealing with CEUS in rectal cancer. Our study showed a positive correlation between enhanced intensity and MVD (*r* = 0.295, *P* = 0.016). Image intensity is proportional to the concentration of bubbles in the vasculature and thus blood flow; increased enhanced intensity showed a tendency toward stronger enhancement and greater perfusion flow, thus correspondent with increased MVD count. We found no association between other CEUS parameters and MVD; this is might be because AT, TTP, WIT, and ascending slope are time-dependent parameters, represented the enhanced speed of the tumor, which might related to spatial distribution of clutter, vascular uneven thickness, distorting, and arteriovenous fistula formation happened in neoangiogenesis, but not number of microvessels. Our findings were very similar to that obtained reported in previous literatures [11, 12, 14]. Therefore, our study suggests that EI could be used for noninvasive estimation of tumor angiogenesis in rectal cancer.

TNM stage and histologic grade are important prognostic factors in rectal cancer. With development of new modalities, additional prognostic indicator for more clinic information may be provided. Some researchers explored the relationship between DCE-MRI perfusion parameters and prognostic factors in rectal cancer, and results were not conclusive. Lollert and Hong reported that DCE-MRI parameters correlated significantly with the N category [31, 32]; Tuncbilek and Hong reported Erise was correlated with N stage, and steepest slope, maximal enhancement, and time to peak were correlated with histologic grade, respectively [32, 33]. On the other hand, Kim found no correlation between any dynamic contrast-enhanced MRI perfusion parameters and TN stage [34]. In our study, enhanced intensity negatively correlated with histologic grade (*r* = −0.295, *P* = 0.007), and none of other parameters correlated with TNM stage and histologic grade. Some research also found significant correlation between MVD and histologic grade of various type of tumors [35–37], suggesting that increased MVD, signifying angiogenesis, is accompanied with higher grade of tumor. Differences in vascularization between well and poorly differentiated tumors might reflect the stromal reaction, interaction of the tumor cells with environments (matrix components, enzymes, and growth factors), and a balance between positive and negative angiogenesis regulators. The process and interaction between tumor cells, endothelial cells, and stroma during tumor progression are very dynamic and determined the tumor growth. At the later stages of tumor progression the angiogenesis was stimulated and tumor cell presented with more aggressive biological behavior. Poorly differentiated tumor cells indicated rapid cell division and thus connote a worse prognosis than well-differentiated tumors [38, 39]. A trend of negative association was seen between EI and T stage. It may suggest that tumor perfusion differed with T staging, but other confounding factors could also contribute to T staging in addition to angiogenesis. The value of CEUS perfusion parameters in indicating prognosis remains to be further investigated.

## 5. Conclusion

In conclusion, enhanced intensity of contrast enhanced endorectal ultrasonography provides noninvasive biomarker of tumor angiogenesis in rectal cancer. CEUS data have the potential to predict patient prognosis.

## Study Limitations

Our study has some limitations. Firstly, the most enhanced region within the tumor from ultrasonography images was drawn as ROI, which might not be correspondent to the hot spot in the histopathological analysis precisely. Secondly, the record time of 60 seconds in CEUS examination was relatively short to include later wash-out phase of perfusion. Thirdly, we did not observe reoccurrence free survival and overall survival rates with a long-term follow-up.

## Figures and Tables

**Table 1:** Correlations of CEUS time-intensity curve parameters with MVD.

CEUS parameters	MVD Count	
	Correlation coefficient	*P*
Arrival time, s	−0.167	0.179
Time to peak enhancement, s	−0.068	0.586
Wash-in time, s	0.025	0.840
Enhanced intensity, dB	**0.295**	**0.016**
Ascending slope	0.071	0.570

CEUS: contrast enhanced ultrasound.

MVD: microvascular density.

Statistical method: bivariate Pearson correlation analysis.

**Table 2:** Correlations of CEUS time-intensity curve parameters with histologic grade and TNM stage.

CEUS parameter	Histologic grade		T stage		N stage		Metastasis	
	Correlation coefficient	*P*	Correlation coefficient	*P*	Correlation coefficient	*P*	Correlation coefficient	*P*
Arrival time, s	0.104	0.287	0.037	0.696	−0.058	0.554	0.022	0.830
Time to peak enhancement, s	0.085	0.332	0.085	0.373	−0.021	0.829	0.058	0.569
Wash-in time, s	0.001	0.995	0.073	0.441	−0.024	0.804	0.018	0.859
Enhanced intensity, dB	**−0.264**	**0.007**	−0.158	**0.096**	−0.026	0.789	−0.018	0.859
Ascending slope	−0.116	0.232	−0.143	0.131	−0.008	0.934	−0.001	0.993

CEUS: contrast enhanced ultrasound.

Statistical method: bivariate Pearson correlation analysis.
